# Surveillance for Influenza Viruses in Poultry and Swine, West Africa, 2006–2008

**DOI:** 10.3201/eid1809.111296

**Published:** 2012-09

**Authors:** Emmanuel Couacy-Hymann, Viviane A. Kouakou, Gilbert L. Aplogan, Felix Awoume, Casimir K. Kouakou, Lamidi Kakpo, Bridgett R. Sharp, Laura McClenaghan, Pamela McKenzie, Robert G. Webster, Richard J. Webby, Mariette F. Ducatez

**Affiliations:** Central Laboratory for Animal Diseases, Bingerville, Côte d’Ivoire (E. Couacy-Hymann, V.A. Kouakou, C.K. Kouakou);; Laboratoire de Diagnostic Vétérinaire et de Sérosurveillance, Parakou, Benin (G.L. Aplogan, L. Kakpo);; Laboratoire Vétérinaire de Lomé, Lomé, Togo (F. Awoume);; and St Jude Children’s Research Hospital, Memphis, Tennessee, USA (B.R. Sharp, L. McClenaghan, P. McKenzie, R.G. Webster, R.J. Webby, M.F. Ducatez)

**Keywords:** influenza, West Africa, temperature, humidity, animal density, viruses, Côte d’Ivoire, Benin, Togo

## Abstract

To determine the extent of animal influenza virus circulation in Côte d’Ivoire, Benin, and Togo, we initiated systematic year-round active influenza surveillance in backyard birds (predominantly chickens, guinea fowl, and ducks) and pigs. A total of 26,746 swab specimens were screened by using reverse transcription PCR. Animal influenza prevalence was estimated at 0 (95% CIs for each of the 2 study years 0–0.04% to 0–1.48% [birds] and 0–0.28% to 0–5% [pigs]). In addition, 2,276 serum samples from the same populations were negative for influenza-specific antibodies. These data indicate that the environments and host populations previously identified as harboring high levels of influenza virus in Southeast Asia do not do so in these 3 countries. The combination of climate and animal density factors might be responsible for what appears to be the absence of influenza virus in the backyard sector of the 3 countries.

## Abstract 2

West Africa might be an animal influenza–free zone.

Relatively little is known about the emergence, prevalence, and circulation of animal influenza viruses in Africa. There is no recent evidence of influenza infection in pigs in West Africa. In 2007, Gaidet et al. (*1*) found a 3.5% prevalence of avian influenza virus in wild birds in Africa; the highest prevalence in Mauritania and Senegal, and the most frequently infected species were Eurasian and African ducks. In addition, low-pathogenic avian influenza viruses of subtypes H1N8, H3N8, H4N2, H4N6, H4N8, H5N1, H5N2, H5N8, H6N2, H7N7, H9N1, and H11N9 have now been detected in wild birds in Nigeria, Egypt, Zambia, and South Africa (*2*–*7*).

Even less is known about avian influenza in domestic poultry in Africa. South Africa has had numerous outbreaks of many distinct influenza subtypes in chickens and ostriches, including H5N2, H5N3, H6N2, H9N2, H10N7, and H6N8 (*3**,**8**–**11*). Egypt is still facing recurrent highly pathogenic avian influenza (HPAI) (H5N1) outbreaks (*12*). In contrast, none of the other affected African countries have reported the pathogen since July 2008 (*13*).

We performed a systematic active surveillance study of animal influenza in Côte d’Ivoire, Benin, and Togo. These 3 West African countries reported cases of HPAI (H5N1) only in 2006, 2007, or 2008 (*13*,*14*). We aimed to confirm the current absence of HPAI (H5N1) from the region and determine whether any other influenza virus strains might circulate in domestic birds and pigs.

## Materials and Methods

### Sampling Sites

Samples were collected exclusively in live-bird markets and backyard farms. The latter were preferred to commercial farms because the outbreaks of HPAI (H5N1) reported during 2006–2008 occurred most often in backyard flocks (for 11/12, 4/5, and 2/3 outbreaks in Côte d’Ivoire, Benin, and Togo, respectively) or on small farms (308–7,771 birds per farm) (*13*,*14*). Sampling sites were selected in the 3 countries for 1) their density of poultry farms (backyard and commercial, even though we focused on the backyard sector, as in the district of Abidjan and in the Middle-Comoé region in Côte d’Ivoire; Lokossa in Benin; Lomé and the Maritime Province in Togo), 2) the presence of water bodies and the possible contact of domestic birds with wild waterfowl (South-Comoé in Côte d’Ivoire; Malanville in Benin); and 3) their past outbreaks of HPAI (H5N1) (district of Abidjan in Côte d’Ivoire; Lomé and the Maritime Province in Togo).

**Figure 1:**
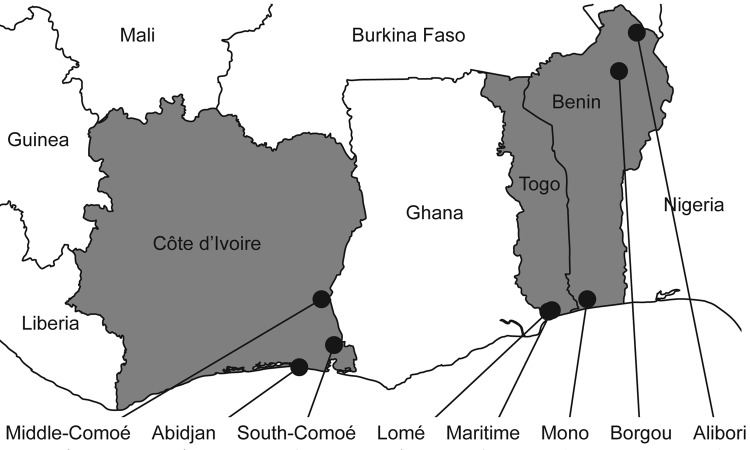
Collection sites of bird and pig samples, West Africa, 2006–2008. Côte d’Ivoire, Benin, and Togo are in gray. Sampling provinces are indicated by black circles.

In Côte d’Ivoire, samples were collected during January 2009–December 2010. Three regions were selected (Figure 1). Specimens were collected in the district of Abidjan (i.e., Bingerville, Marcory, Treichville, Port-Bouet, Koumassi, Yopougon), the Middle-Comoé region (i.e., Agnibilékro, Takikro, Abengourou, Niablé), and the South-Comoé region (i.e., Aboisso, Adiaké). In Benin, samples were collected during November 2008–September 2010 in live-poultry markets in Malanville, Gogounou, and Dérasi in the provinces of Borgou and Alibori in the north of the country (Figure 1). A total of 200 swab samples were collected from birds in Lokossa (Mono Province) in 2009. The specimens from pigs were collected from animals in slaughterhouses in Parakou (Borgou Province).

In Togo, swab specimens from birds were collected in January and March 2009 and during February–December 2010. Locations were live-poultry markets in Adidogomé, Aklakou, Tabligbo, Vogan, Agoé, Akodesséwa, Aného, Tsévié, Adawlato, and Gbossimé, in Lomé and in the Maritime Province (Figure 1).

In each region in Côte d’Ivoire, a minimum of 5 villages were randomly selected among those willing to participate. Birds from live-bird markets were randomly selected before sampling (5 randomly selected birds per vendor, number of vendors randomly selected depending on the total number of specimens to collect in a given market).

### Sample Collection

At each sampling site, >25 birds were clinically examined, and tracheal and cloacal swab samples were collected at least monthly. In Côte d’Ivoire, nasal swab samples from pigs were collected monthly in 2009 and every 3 months in 2010. In backyard flocks in Côte d’Ivoire, serum was collected every 3 months. Each selected market was visited 1×/month in Togo and 2×/month in Benin.

The samples were collected in viral transport media as described (*15*) and then stored in liquid nitrogen or on ice during sampling and transportation to the laboratory, which never exceeded 1 day. Swabs were then immediately stored either at –80°C in Côte d’Ivoire and Benin or in liquid nitrogen in Togo before further processing. Serum was stored at –20°C before further processing.

### Serologic Testing

Serum was screened for influenza antibodies by performing ELISAs and/or hemagglutination inhibition (HI) assays. ELISAs were performed by using the FlockChek AI MultiS-Screen Ab Test Kit (Idexx, Westbrook, ME, USA) according to the manufacturer’s instructions. HI assays to detect influenza virus were performed as described (*15*,*16*) by using inactivated H5 (A/whooper swan/Mongolia/244/05), H6 (A/turkey/Massachussetts/65), H7 (A/ruddy turnstone/New Jersey/65/85), and H9 (A/duck/Hong Kong Special Administrative Region, People’s Republic of China/Y280/97) antigens and positive- and negative-control serum. Serum samples were screened for Newcastle disease virus (NDV)–specific antibodies by performing HI assays with inactivated reference antigen and positive- and negative-control serum.

### Molecular Testing

Tracheal and cloacal swabs were processed as described (*17*,*18*). The samples were screened either individually or in pools of 2 or 5 swabs. RNA was isolated by using the RNeasy mini kit (QIAGEN, Valencia, CA, USA), the QIAmp viral RNA minikit (QIAGEN), or the MagMAXTM-96 AI/ND viral RNA isolation kit (Applied Biosystems/Ambion, Austin, TX, USA) with a Kingfisher Flex magnetic particle processor (Thermo Scientific, Rockford, IL, USA). RNA was eluted in 50 μL of nuclease-free water.

The swab samples from Côte d’Ivoire were tested by using 2-step reverse transcription PCRs (RT-PCRs). The RT step was performed by using random hexamers (Invitrogen, Carlsbad, CA, USA) with 10 μL of extracted RNA and the First-Strand cDNA Synthesis kit (GE Healthcare Europe GmBH, Orsay, France,) according to the manufacturer’s protocol. Next, 5 µL of the cDNA obtained was used as the template for the PCR step. The PCR was performed by using the Gene Amp PCR System 2400 (Perkin-Elmer, Applied Biosystem, Paris, France) as described (*14*).

The swabs from Benin and Togo were tested by using 1-step RT-PCRs performed with the Qiagen 1-step RT-PCR kit (QIAGEN) with either an ABI 9700, ABI 2720 (Applied Biosystems, Vienna, Austria) or ABI 7500 (Stratagene; Applied Biosystems, Carlsbad, CA, USA) thermocycler. For conventional RT-PCR screenings, we used primers that target the influenza A matrix gene (*19*) and the following cycling conditions: 1 cycle of 50°C for 30 min; 1 cycle of 95°C for 15 min; 40 cycles of 95°C for 30 sec, 60°C for 30 sec, and 72°C for 1 min; and 1 cycle of 72°C for 10 min. For real-time RT-PCR screenings, we used influenza A primers (*18*) with the following cycling conditions: 1 cycle of 50°C for 30 min; 1 cycle of 95°C for 15 min; and 40 cycles of 95°C for 10 sec and 60°C for 30 sec.

Swab samples from birds were screened for other avian pathogens. We used PCR to screen for DNA viruses (infectious laryngotracheitis [ILTV], Marek’s disease virus [MDV], and chicken anemia virus [CAV]) and 2-step RT-PCR to screen for RNA viruses (NDV, infectious bronchitis virus [IBV], avian metapneumovirus [aMPV], and infectious bursal disease virus [IBDV]) as described (primers available on request).

## Results

### Sample Collection

We collected 25,136 swab and 1,819 serum samples from birds and 1,610 swab and 457 serum samples from pigs during the 2-year survey in the 3 countries. Of the bird samples, 70% were from live-poultry markets and 30% from backyard flocks (Table 1 and Table 2). Specimens were collected year-round, and monthly samples ranged from 20 to 160 and from 218 to 1,778 per month, from swine and poultry respectively.

**Table 1:** Active surveillance for animal influenza, West Africa, November 2008–December 2010

Country	Bird samples tested, no				Pig samples tested, no.	
	Oropharyngeal swab	Cloacal swab	Serum		Nasal swab	Serum
Benin	5,230	4,959	100		62	0
Côte d’Ivoire	6,240	6,253	1,283		1,548	457
Togo	1,149	1,305	436		0	0

**Table 2:** Bird species and collection sites in surveillance for animal influenza, West Africa, November 2008–December 2010

Country	Bird species, %				Type of collection site (% of samples collected)*
	Chicken	Guinea fowl	Duck	Pigeon	
Togo	70	11	13	6	LBM (100)
Benin	88	11	0.001	0.002	LBM (100)
Côte d’Ivoire	92	1	7	0	LBM (34), BB (66)
Total	88	7	5	0.5	LBM (70), BB (30)

*LBM, live-bird markets; BB, backyard birds.

### RT-PCR Screening

**Figure 2:**
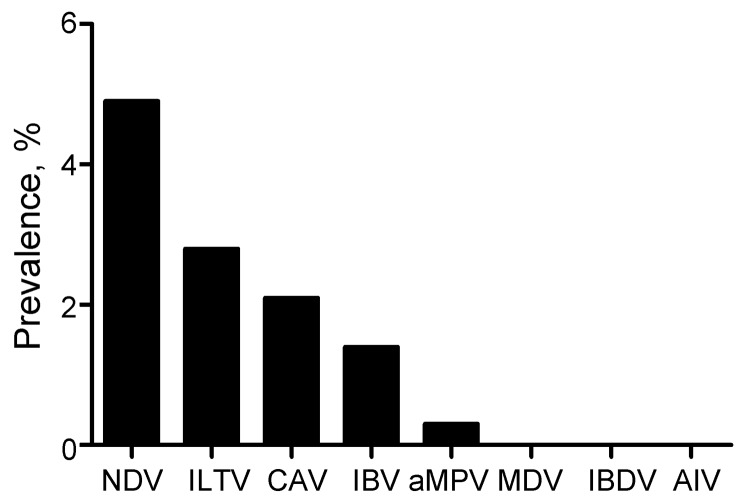
Prevalence of 8 avian viruses detected by reverse transcription PCRs of a subset of 2,427 tracheal and cloacal swab samples collected in live-bird markets, Benin and Togo, 2009. NDV, Newcastle disease virus; ILTV, infectious laryngotracheitis; CAV, chicken anemia virus; IBV, infectious bronchitis virus; aMPV, avian metapneumovirus; MDV, Marek’s disease virus; IBDV, infectious bursal disease virus; AIV, avian influenza virus.

The 26,746 total swab samples collected from birds and pigs in Côte d’Ivoire, Benin, and Togo all tested negative for influenza A genome by RT-PCR, irrespective of collection month or host, and the annual prevalence per country was null (95% CI 0.04–4.79%) (Table 1, Table 2, Table 3). To verify that cold-chain or storage problems had not simply degraded our samples’ nucleotides, we screened a subset of 2,427 swab samples collected from birds during early 2009 and 2010 from Benin and Togo for other RNA avian viruses (NDV, IBV, IBDV, or aMPV) and DNA viruses (CAV, ILTV, or MDV). Of the 2,427 samples collected in Benin and Togo, the prevalence of the other viral pathogens ranged from 0 for MDV and IBDV to 4.9% for NDV (119 positive samples), 2.8% for ILTV (68), 2.1% for CAV (51), 1.4% for IBV (34), and 0.3% for aMPV (7) (Figure 2). In addition, 3,330 swab samples collected from birds in Côte d’Ivoire in 2010 were screened for NDV; NDV prevalence ranged from 0.3% to 1.4% depending on time (data not shown). Taken together, these results show the fair quality of our specimens. Cold-chain and sample quality were unlikely to account for the absence of detected influenza virus RNA.

**Table 3:** Prevalence of animal influenza, West Africa, 2009–2010

Year	Country	Animal*	Specimens tested, no.	Prevalence, % (95% CI†)
2009	Côte d’Ivoire	Bird	3,895	0 (0–0.08)
	Côte d’Ivoire	Pig	62	0 (0–4.79)
	Benin	Bird	5,669	0 (0–0.06)
	Benin	Pig	1,112	0 (0–0.28)
	Togo	Bird	204	0 (0–1.48)
2010	Côte d’Ivoire	Bird	8,598	0 (0– 0.04)
	Côte d’Ivoire	Pig	436	0 (0–0.7)
	Benin	Bird	3,720	0 (0–0.09)
	Togo	Bird	2,250	0 (0–0.14)

*Backyard and live-bird market poultry. †95% CIs were calculated with a sensitivity of 99% and a specificity of 100% for the reverse transcription PCR.

### Serologic Testing

Because influenza virus infection might last only a few days in birds and pigs, we could have missed the virus in the animals sampled. Therefore, we conducted serologic screening, which provides insight into the infection history of an animal’s entire life. None of the serum samples collected in birds in Côte d’Ivoire, Benin, and Togo were positive for influenza antibodies by ELISA or HI assay. Although 16 of 457 pig serum samples from Côte d'Ivoire were weakly positive by ELISA, none were confirmed positive by HI; they most likely were all negative for influenza antibodies. NDV antibodies were detected in 20% of serum samples from birds in Côte d’Ivoire and in 32% of bird serum samples from Togo (data not shown).

### Influenza Seasonality and Environmental Factors

Determining the factors contributing to the seasonality of influenza has been difficult because some countries report having influenza activity year-round and others report having 2 peaks of activity or a combination of these patterns. To follow up on recent results (*20*) seemingly confirming the year-round activity hypothesis that states that influenza spread in the tropics is due to contact rather than aerosol transmission, we compared the average livestock production, temperature, and relative humidity (RH) levels in Côte d’Ivoire, Benin, and Togo with those of Nigeria, Egypt, Vietnam, and Indonesia. Côte d’Ivoire, Benin, and Togo produce significantly less bird and pig meat and fewer bird eggs than do Nigeria, Egypt, Vietnam, or Indonesia (Table 4). West Africa is hot and humid all year, with temperatures ranging from 22°C to 32°C and RH ranging from 63% to 82% (Table 4).

**Table 4:** Role of influenza virus stability, as influenced by temperature and relative humidity, on hen egg and livestock production, Benin, Côte d’Ivoire, Egypt, Nigeria, Togo, Indonesia, and Vietnam*

Country	Livestock production					Temperature, °C (range)¶#	Relative humidity, % (range)#**
	Hen eggs†‡	Chicken meat‡§	Turkey meat‡§	Duck meat‡§	Pork‡§		
Benin	306	22	ND	ND	4	26.3 (23–30)	74.9 (70–81)
Côte d’Ivoire	610	23	ND	ND	7	26.7 (22–32)	75.3 (70–82)
Egypt	7,000	629	10.5	39	2	22 (9–35)	35.2 (35–46)
Nigeria	12,284	243	ND	ND	218	26.4 (21–33)	84.7 (80–88)
Togo	174	9	ND	ND	9	26.6 (22–32)	70.6 (63–78)
Indonesia	1,123	1,450	ND	31	637	27.7 (23–33)	80.6 (75–85)
Vietnam	247	448	ND	82	2,470	24.1 (13–33)	71.1 (67–76)

*Livestock production: Food and Agriculture Organization data for 2008 (*21*), temperature and relative humidity data: www.climatetemp.info/, March 28, 2012. ND, no data. †Combination of official data, Food and Agriculture Organization estimates, and calculated data. ‡× 10^6^ eggs. §× 10^6^ kg. ¶Annual mean value (coldest monthly average low temperature to warmest monthly average high temperature). #Temperature and relative humidity were measured in the following cities: Cotonou, Benin; Abidjan, Côte d’Ivoire; Cairo, Egypt; Lagos, Nigeria; Lomé, Togo; Jakarta, Indonesia; and Hanoi, Vietnam. **Annual mean value (driest monthly relative humidity average to most humid monthly relative humidity average).

## Discussion

HPAI (H5N1) was detected in West Africa during February 2006–July 2008 (*13*,*14*), and all the strains characterized belonged to clade 2.2 (*22*,*23*). Whether wild birds or trade brought the virus to Africa remains unclear (*24*–*26*). However, evidence suggests that the pathogen was first detected in People’s Republic of China and might have transited through western Asia, Russia, or Europe (*27*), with wild birds probably playing a role in introducing HPAI (H5N1) to Africa (*27*–*31*). Incidentally, Côte d’Ivoire, Benin, and Togo are on the Black Sea–Mediterranean and East Atlantic flyways that these birds use for migration (*32*).

Within a year after the initial outbreaks, the clade 2.2 strains were thought to have become endemic in Nigeria, and intra–clade 2.2 reassortant viruses were characterized, highlighting the effects of the virus virus and its evolution within the country (*33*,*34*). Nigeria has not reported an HPAI (H5N1) outbreak since July 2008 and now seems free of the pathogen (*13*). The neighboring countries reported only isolated outbreaks in 2006, 2007, or 2008, but no sustained transmission has been reported (*13*).The subtype H5N1 outbreaks in backyard poultry in West Africa were associated with less severe symptoms and lower death rates than those usually described in such outbreaks.

That HPAI (H5N1) is endemic in several Southeast Asian countries and in Egypt but did not persist in West Africa, except for a couple of years in Nigeria, is intriguing. Despite the effect of viruses such as NDV, influenza virus was not detected in any of the swabs or serum samples collected during our active surveillance for animal influenza in Côte d’Ivoire, Benin, and Togo. Several factors, such as type of hosts available, animal density, and climate with its effect on virus persistence in the environment and on virus transmission, might have prevented continued circulation of the virus in the region.

Ducks in particular are a natural reservoir for influenza and play a major role in influenza transmission (*35*). Fewer ducks are raised in Africa than in Southeast Asia (Table 4), which might limit the virus pool and sustainability on the continent. Moreover, the few ducks in West African are backyard birds in low density flocks, not free-range birds in large flocks on lakes and rice paddies as they often are in Southeast Asia. Chickens have so far been the first host infected by subtype H5N1 in Egypt (*36*), but duck meat production is much higher in Egypt than in Côte d’Ivoire, Benin, Nigeria, or Togo (Table 4). The structure of live-bird markets in West Africa also differs substantially from that in Southeast Asia, with fewer birds, fewer species (large majority of chickens and guinea fowls), and often lower confinement (authors’ observations).

We believe that temperature and humidity might be critical parameters for the survival of influenza virus in West Africa. Temperature and humidity affect the duration of virus persistence in the environment. In the guinea pig model, influenza transmission by the aerosol route depends on humidity and temperature, although contact transmission does not (*37*,*38*). Of the experimental conditions tested, only 5°C, 20°C, and 35% RH allowed 100% aerosol transmission; thus, warmer and more humid environments might have less aerosol transmission of influenza virus (*37*). Côte d’Ivoire, Benin, Nigeria, and Togo are hot and humid countries year-round. Therefore, one would expect shorter virus persistence in the environment, and, according to the data obtained by using the guinea pig model, one would expect contact transmission rather than aerosol transmission of influenza in West Africa. Still, aerosol transmission may occur in Egypt because the RH is much lower than in West Africa and the temperature drops to 9°C in the winter. However, Vietnam and Indonesia (2 countries to which HPAI [H5N1] outbreaks are endemic) are just as hot and humid year-round as West Africa, yet subtype (H5N1) is maintained in birds. Thus, the climate cannot be the only factor limiting influenza in West Africa.

We considered an additional factor—animal density—while trying to determine why influenza might not be sustained in West Africa. The amount of eggs and bird meat and pork produced is considerably lower in Côte d’Ivoire, Benin, Nigeria, and Togo than in Egypt, Nigeria, Vietnam, and Indonesia. Thus, we hypothesized that a high animal density might be required for sustained transmission of the virus. This high density would explain why subtype H5N1 seems to have persisted in Nigeria, with its large avian commercial sector, for a couple of years while causing only sporadic outbreaks in neighboring countries. Our hypothesis is in agreement with the recent finding that influenza prevalence in Egypt is higher in commercial flocks than it is in backyard flocks (*36*).

If influenza virus transmission is limited by climate and animal density, then these limitations should apply to other similar pathogens. In that case, the high prevalence of NDV in Benin and Togo is surprising. However, we had a sampling period bias because all of the NDV (and noninfluenza virus) from screened specimens from Benin and Togo was from specimens collected during January–March when NDV is known to cause disease in the western African backyard sector. Moreover, Songer et al. showed that NDV aerosol transmission at 23°C is better at 10% RH than at 90% RH or 35% RH (*39*). Although we lack data on whether NDV transmission differs from influenza transmission, different viruses, even those with the same nucleic acid core, might have different sensitivities to aerosol generation, depending on the RH level (*39*). Further experiments are warranted to determine whether NDV and influenza A virus transmission patterns actually differ and whether temperature and humidity have any role in that process.

We cannot exclude the possibility that influenza might exist in areas that we did not check. Such influenza hot spots have been discovered for influenza, including 1 at Delaware Bay in the United States (*40*). The requirements for continued circulation of influenza virus in animals (and the role of domestic animals in maintenance and interspecies spread) are not well understood. Future surveillance in West Africa should include more collection sites and include the commercial sectors and wild bird population to survey putative faster transmission and new introductions.

Our systematic year-round active influenza surveillance program in the backyard sector in Côte d’Ivoire, Benin, and Togo showed a prevalence of 0 (95% CI 0–0.04% to 0–1.48% in birds and of 0–0.28% to 0–5% in pigs). We hypothesize that the combination of climate and animal density factors might be responsible for what appears to be the absence of influenza virus in the backyard sector of the 3 countries we studied.
